# Evolution of Complexity. Molecular Aspects of Preassembly

**DOI:** 10.3390/molecules26216618

**Published:** 2021-10-31

**Authors:** Fredric M. Menger, Syed A. A. Rizvi

**Affiliations:** 1 Department of Chemistry, Emory University, Atlanta, GA 30322, USA; 2 School of Pharmacy, Hampton University, Hampton, VA 23669, USA; syed.rizvi@hamptonu.edu or

**Keywords:** complexity, echolocation, epigenetics, non-coding genes, preassembly, evolution theory

## Abstract

An extension of neo-Darwinism, termed preassembly, states that genetic material required for many complex traits, such as echolocation, was present long before emergence of the traits. Assembly of genes and gene segments had occurred over protracted time-periods within large libraries of non-coding genes. Epigenetic factors ultimately promoted transfers from noncoding to coding genes, leading to abrupt formation of the trait via de novo genes. This preassembly model explains many observations that to this present day still puzzle biologists: formation of super-complexity in the absence of multiple fossil precursors, as with bat echolocation and flowering plants; major genetic and physical alterations occurring in just a few thousand years, as with housecat evolution; lack of precursors preceding lush periods of species expansion, as in the Cambrian explosion; and evolution of costly traits that exceed their need during evolutionary times, as with human intelligence. What follows in this paper is a mechanism that is not meant to supplant neo-Darwinism; instead, preassembly aims to supplement current ideas when complexity issues leave them struggling.

## 1. Introduction

Echolocation systems in bats (Chiroptera) are complex “organs of perfection” enabling bats to locate and devour tiny, evasive insects in night-time flight [1,2,3,4,5,6,7,8,9]. It is an example of the complexity in biology whose evolution is the focus of this paper. Only a portion of the >1000 bat species possess this skill. Toothed whales also make use of “biological sonar” but, since the two animals are not in any way related, their traits must have evolved by convergent evolution. The oldest known bat fossil dates back to the Eocene period about 52 million years ago [10,11]. Clearly recognizable as a bat, this fossil put to rest a long-standing debate over which came first, flight or echolocation. The ancient bat was able to fly but made no use of echolocation, a fact evident from ear bones that were not enlarged as they are in modern bats that use echolocation. Early bats seem to have been diurnal, using only their eyes to navigate. Presumably bats were forced to become nocturnal upon appearance of avian predators, sometime soon after dinosaurs became extinct. The origins of bat species are poorly understood although evolutionists believe that mammalian flight most likely evolved among arboreal locomotors (“trees-down”) rather than among terrestrial runners (“ground-up”) [12]. Evolution of echolocation, per se, remains largely a mystery.

Philip Ball in his “Stories of the Invisible” wrote that a genetic mutation can have a beneficial effect, although “the advantage might be extremely slight” [13]. He goes on to write that “evolution advances through such infinitesimal steps, as tiny advantages lead to fractionally higher reproductive success, and thus to a slow increase in the incidence of the mutated gene in the population” [13]. These words express neo-Darwinism’s main thesis. Clearly, the theory demands the presence of innumerable intermediates as one organism transforms into another. Yet, there exists no fossil evidence of creatures in the process of developing echolocation in a smooth continuous sequence. Rudimentary echolocation abilities are indeed present in shrew, rats, and swiftlets [14,15]. Even some blind humans are thought to have radar-like abilities [16], but their evolutionary import seems tenuous in the absence of these animals having echolocation anatomy and neurology even remotely resembling those in bats. Thus, there exists no fossil evidence of creatures with rudimentary echolocation in the process of becoming, step-by-step, the sophisticated systems found in bats. Absence of such fossils might, possibly, be attributed to the fact that the trait in question was “soft” and did not fossilize well. Be that as it may, no gradual improvements in echolocation development, from simple to complex and consistent with neo-Darwinism, have been found in Nature, past or present. Insights into the morphological diversity of modern bat skulls have probably been the closest evolutionists have come to addressing the mystery of bat lineages [17]. It is concluded therefrom, somewhat vaguely, that the dynamics of skull shape macroevolution in bats are best described by a “series of discontinuous shifts that correspond to dietary ecology and sensory adaptations” [17].

The next three paragraphs will present an overview of bat echolocation anatomy [1,2,3,4,5,6,7,8,9]. Its purpose is not so much to set forth the details of this organ structure as it is to illustrate the exquisite complexity of the trait. Complexity here is defined as an intangible mixture of multiple genes and traits that define a structure. After scanning this information, the reader is challenged to ponder how echolocation evolved in “infinitesimal steps” according to neo-Darwinian doctrine. Bear in mind that each step must represent a positive shift toward reproductive success. In contrast to echolocation, evolution of the human eye, with its many living and fossil gradations from simple to complex, lends itself more readily to conventional thought.

Some bats use their larynx to produce ultrasonic waves that are emitted through the mouth or nose. Other bats produce clicks with their tongues. Sound is projected by a wrinkled fleshy nose that acts as a type of megaphone. The delay and frequency of sound waves bouncing back from objects hit by the sound waves inform the bat as to the speed, direction, size, and distance of the prey. A piece of skin in the front of the ear canal, the tragus, directs sound into the ear. Just before a bat emits a sound, tiny bones in the inner ear separate so that the bat’s hearing is not damaged. Once the larynx muscle has contracted, the middle ear relaxes, allowing the bat to hear incoming echoes. To help avoid deafening themselves on emitted sounds that can reach a remarkable 120 decibels, bat signals fall outside the bat’s audible frequency range. This works well because echoes have a lower frequency detectable by the bat. A single echolocation call can last anywhere from 0.2 to 100 ms in duration depending on the proximity of the prey. The time intervals between subsequent calls are typically 100 ms for a bat searching for insects, but can decrease to 5 ms in the final moments of capture [10].

The auditory systems of bats are particularly amazing. The key organ is the cochlea, a hollow spiral-shaped bone in the inner ear that converts sound vibrations into nerve impulses with the aid of hair cells. A specialized basilar membrane within the cochlea responds to the frequency of returning echoes. For example, the basilar membrane of the horseshoe bat has a disproportionate lengthening and thickening that responds to 83 kHz, the common frequency of the bat’s echo. Bat ears can detect even tiny frequency modifications created by the echoes. Sensitivities of bat echolocation also rely on an important protein called prestin. Prestin is a type of biological motor functioning at microsecond rates, orders of magnitude faster than any other cellular motor protein, at the outer hair cells of the inner ear. Information processing in the auditory cortex of the echolocator’s brains is also highly specialized in a manner not found in non-echolocating animals. There are even “delay-tuned” neurons in the cortex that respond to a specific time-delay between the call and echo. Other neurons, called combination-sensitive neurons, respond to specific combinations of frequency and timing. Among the dozens of genes known to be involved in the auditory perception system, twelve are engaged in bone formation and are thought to enable bats to hear high frequencies [10].

**Figure 1:**
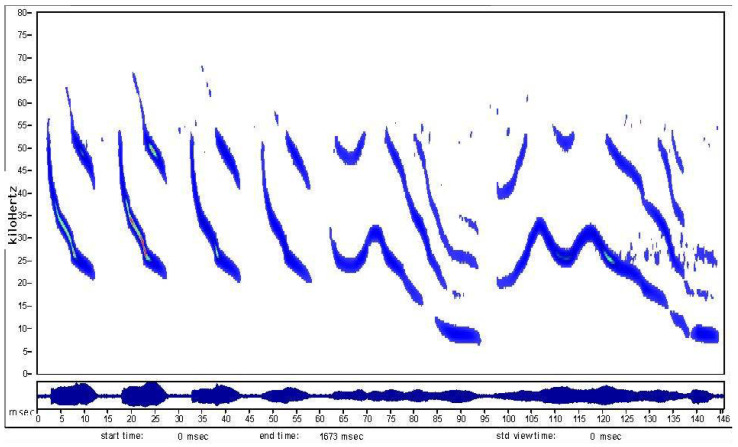
In this recording, the pallid bat (Antrozous pallidus) makes four echolocation calls followed by social calls. The spectrogram above (kHz vs. msec) is a graphic representation of the sounds. Credit: Katy Warner, Colorado State Univ.

The sophistication of echolocation capabilities can be further revealed by citing the Brazilian free-tailed bat. When flying near the ground, the bats use shorter, higher frequency and broader bandwidth calls compared with the same bats flying at higher altitudes [18]. Altitude-sensitivity in bats is useful owing to the greater levels of echo-producing clutter (trees, buildings etc.) found near the ground. The complexity of echolocation is also seen in the following published statement: “Nasal emitting bats are endowed with a variety of both hard and soft tissue adaptations including bony nasal domes, frontal concavities, floating premaxillae, modified turbinals, and elaborate nose leaves” [19]. One need not necessarily understand what role these structures play, or the genetics behind them, to appreciate the astounding complexity of echolocation (Figure 1).

The total number of genes that a bat needs to create echolocation, and to guide its operation, is unknown. A rough, order-of-magnitude estimate is, however, possible by examining another “organ of perfection”, the eye, for which more information is available. Thus, an exhaustive study by the International Mouse Phenotyping Consortium evaluated 4364 mouse genes and identified 347 genes that influence ocular phenotypes [20,21]. Twenty-four gene-mutations, for example, suffice to cause myopia. And about 1000 human genes are known to direct our olfaction receptors [22]. Thus, hundreds of genes are no doubt involved with echolocation, leaving evolutionists with the unanswered question, “How did they arise?” In particular, clarification is needed regarding the time-sequence by which each component within the intricate structure made its individual appearance. Although no one can supply this essential information at the present time, it is possible to view the evolution of complexity in more general terms, and this is undertaken in the next section.

## 2. Discussion

With the preceding background information in hand, let us now contrive a neo-Darwinian story describing how echolocation came into being. This narrative begins with a primitive echolocation trait being already in place within a single prehistoric bat, thereby contributing to its fecundity and allowing the trait to spread throughout the population. Note that it was necessary for the story to propose a primitive echolocation capability from its very beginning. Otherwise, natural selection would obviously be unable to have promoted the occurrence of the trait. Apart from the difficulty of constructing a primitive, yet, functioning echolocation anatomy, other problems with the neo-Darwinian story are encountered. In particular, how did echolocation, albeit a primitive form of it, originally appear among a population of bats totally lacking the trait? A single new mutation, corresponding to one new protein, cannot easily be imagined as sufficient to create even a rudimentary echolocation ability. A large but unknown number of concurrent mutations is likely needed to achieve even the simplest echolocation system. But mutations are rare and mainly harmful, so that an extended time-period must ensue before an entire family of mutations is ultimately acquired. Yet, in a large population, the bat that receives the first echolocation mutation is statistically unlikely to be the bat that receives the second mutation or, for that matter, any of the others. Consequently, the large number of echolocation genes would be distributed among an equal number of bats, none of whose genes would, by themselves, provide a functioning echolocation, however primitive. Echolocation would appear only if each gene disseminated into the population where it could mix efficiently and operate in concert with other related echolocation genes. In other words, the echolocation trait would take hold only if individual echolocation genes (whose initial stages are of no import to the bat) could nonetheless survive sufficiently long over a protracted time-period required by an excruciatingly slow series of “neutral” mutations.

To restate the problem faced by the neo-Darwinian scenario: Prior to the individual genes gathering into an intact “echolocation cluster,” the initial genes are for all intents and purposes assumed to be useless. But if the newly formed genes failed to manifest any benefit, then there was no obvious bio-criteria by which natural selection could favorably screen them. The neo-Darwinian “one-tiny-mutation-at-a-time” mechanism leaves one perplexed as to how early echolocation mutations (genes that could not impart echolocation by themselves) would have been spread across the population by natural selection. Bear in mind a basic tenet of neo-Darwinism: the mechanism is not predictive of future capabilities. Natural selection would not have fostered genes whose utility manifested itself only eons after the genes’ actual appearances.

One might, of course, claim that the early echolocation genes, destined to eventually become useful to bats in their pursuit of insects, had peripheral purposes unrelated to echolocation. Each of Phillip Ball’s “infinitesimal steps” [13] in the early evolution of echolocation had, according to this line of thinking, secondary functions unrelated to echolocation. These secondary functions allowed a natural selection process to take place when this would not be feasible based on echolocation genetics alone. In other words, secondary factors “dragged” echolocation along with them. It is difficult to comment on this rationale because, for one reason, possible secondary functions in echolocation evolution have not been established. Let it simply be stated here that attributing an unknown pathway to the success of another unrelated pathway, also unknown, seems nonproductive.

A neo-Darwinian mechanism states that bat echolocation evolution continued unabated until hundreds of mutations, each of them beneficial to the cause, were gathered in at least one individual. This brings up another drawback to the construct—time—as illustrated by two questions. (1) How long must a proposed intermediate wait until it is modified (beneficially, of course) by rare, random, and usually harmful mutational forces? Note that this question does not ask for general mutation rates but, instead, the probability of an extended series of specifically needed mutations occurring in exactly the correct sequence. (2) How long will it take for natural selection to expand the presence of new mutations throughout a population, especially if the mutation is only mildly beneficial within slowly reproducing and migrating animals? Since a minimum of several hundred mutations are associated with echolocation, the time requirements for these all these mutation/mixing events would have been immense.

A crude sense of the time requirements can be obtained from the Genetics publication of Durrett and Schmidt who calculated the waiting time for a pair of pre-specified mutations [23]. They selected for their model a Drosophila mutation that inactivates a transcription factor waiting for a second mutation that reestablishes the trait. The results, which are strongly dependent upon a series of reasonable assumptions (concerning nucleotide mutation rate, population, neutrality of mutations etc.), show that the second specific mutation appears after a wait of 9 million years! If the second mutation is mildly advantageous, then the waiting time decreases to 400,000 years. The point of this is not to provide exact numbers, as much as to show that an evolution advancing in “infinitesimal steps” is horrendously slow, especially during early stages when mutations are “neutral”, i.e., they provide no survival advantage. Furthermore, bear in mind that the even greater unlikelihood is associated with a necessary coordinated third (not to mention hundredth) specified mutation. Note that small populations, which are more susceptible to higher rates of natural selection and genetic drift, should not be called upon to invoke shorter evolutionary times, because small populations also seriously impair the already meager likelihood of specific beneficial mutations appearing at each step in the evolution.

Evolutionists are well aware of the statistical problems just mentioned. In fact, it is for this reason that neo-Darwinism must assume a myriad of tiny accessible steps, each of them being increasingly profitable to the organism [13]. But the lack of discrete intermediates in echolocation, demanded by this mechanism, discredits such a proposal. Additionally, such examples are given in the next paragraph.

**Figure 2:**
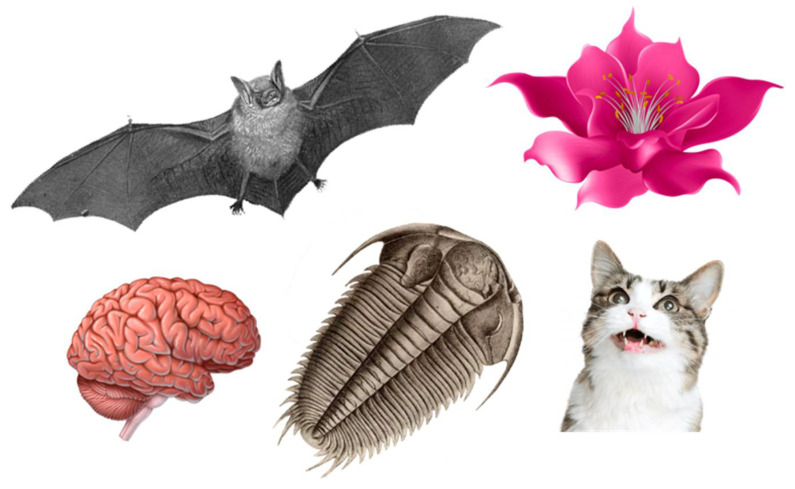
Pictures representing bat echolocation, flowering plants, human intelligence, trilobites, and cat domesticity whose evolutions all defy the neo-Darwinian model.

Examples of evolutionary changes at odds with the conventional neo-Darwinism have been published previously [24,25]. These will now be mentioned, but only cursorily, in further support of the notion that neo-Darwinism cannot be the whole story. (1) Humans are now far more intelligent than required for their hunting-gathering >10,000 years ago (unless living-off-the-land activities of our ancestors are equated with solving differential equations, writing symphonies, designing computers, or even driving an automobile). From where, therefore, did our intelligence derive? (2) Domestication of wildcats into housecats, a process that involved multiple genetic and physical modifications, took place in less than 12,000 years. This is a trivial time period relative to both mutational and evolutionary time-scales. (3) During the so-called Cambrian explosion, about 540 million years ago, a period of incredible diversity of life began. Most currently known body plans first emerged during this time. Yet fossils of ancestors to Cambrian life cannot be found in pre-Cambrian rock, in opposition to what is expected from neo-Darwinism. S. J. Gould once commented that “The Cambrian explosion was the most remarkable and puzzling event in the history of life” [26]. (4) Flowering plants appeared abruptly in the Cretaceous period, with fossil records showing no previous forms related to these angiosperms. Darwin himself called flowering plants an “abominable mystery” (Figure 2) [27,28].

Echolocation and the four additional evolutionary riddles are far too widespread, diverse, and profound to be casually dismissed as “anomalies” or “rare exceptions”. A more broadly based evolutionary theory, one that includes a faster and less “piecemeal” structure-development, is called for. Such a speculative theory is about to unfold. Theological notions of evolution, incidentally, will not be included in the discussion as they belong to a separate philosophic domain.

Before proposing an alternative to neo-Darwinism in the next section, an important point must be made absolutely clear. The intent here is to supplement neo-Darwinism, not supplant it. Once echolocation was established on earth, then it stands to reason that conventional neo-Darwinism served to fine-tune the system as it does, for example, with bacteria becoming penicillin-resistant. Such alterations, however, fall under the category of “microevolution” and involve simple one gene/one enzyme modifications. Alternative models are needed especially with the complex systems, such as those just cited, where neo-Darwinism is deficient in explaining large numbers of interconnected modifications for which relevant intermediates are absent.

## 3. Preassembly

Little gain is achieved by merely citing deficiencies in a current theory without also offering, in its place, a better model. The history of science tells us that a theory is often retained, despite recognized inconsistencies, until such time that another theory lacking those inconsistencies becomes available [29,30]. Thus, what follows is an alternative to neo-Darwinian theory intended to address those cases where the classical theory is deficient. These include echolocation and several other examples, as previously mentioned. A speculative alternative view of neo-Darwinism, designated “preassembly”, avoids difficulties that trouble the current model. The next section of this paper focuses on the preassembly as a component of modern evolutionary thought.

Preassembly is a theory postulating that much of the genetic information necessary for the evolution of certain complex traits had been “prepackaged” and available at the time the traits began appearing. Hence, the term “preassembly”. Many puzzling observations in evolution become more understandable with the aid of this one single idea. Consider echolocation. In early evolutionary times, tens of millions years ago before the appearance of echolocation in bats, genetic material was forming and accumulating as part of the bats’ non-coding DNA library. This library served as a repository of change. In humans, 98% of the DNA is non-coding (incorrectly referred to as “junk DNA” [31]), sufficient to create a million genes. Details of pre-echolocation bat genetics are, of course, unknown and perhaps unknowable. But one could imagine that potentially useful genes, and relevant gene segments, were being randomly and silently collected and retained (without selection) in a huge non-coding DNA assembly. Among these were echolocation genes, and their gene precursors, which were eventually released by epigenetic factors when bats were forced into nocturnal living [32,33,34]. Additionally, consider the Cambrian explosion during which a rich variety of life evolved with no fossil evidence of precursors [25]. Preassembly assumes that the genetic wherewithal for these extraordinary new life-forms had been accumulating in the Ediacaran era that preceded the Cambrian for 100 million years. With the climatic changes of the Cambrian era, partially or fully formed genes began to be incorporated into new animal life-forms. The process was amazingly abrupt (hence being called an “explosion”) because genetic support for the diversity had been, at least partially, already in place. Clearly, evolutionary speed is an important attribute of preassembly.

Preassembly invokes a vast collection of assorted non-coding genes most of which are never to be exploited. Given proper epigenetic stimuli [32,33], useful de novo genes and gene segments among this library began to express themselves, i.e., manifesting a noncoding-to-coding shift, and new traits would appear. The construct has its difficulties, to be discussed momentarily, but at the very least the theory allows one to disregard the idea of complex organs evolving via those “infinitesimal external steps” as proposed in neo-Darwinism. Moreover, the vast time-periods associated with hundreds of rare, specific, and sequential mutational events, as would be required by a neo-Darwinian-based echolocation, also become moot. This is because genetic transformations over the ages had already taken place randomly and silently within non-coding DNA. In short, several difficulties with the neo-Darwinian model referred to above are averted in the preassembly mechanism.

The preassembly model states that rapid evolution arose in part from ancestral, non-coding DNA sequences [35,36,37,38,39]. The sequences transformed into de novo protein-coding genes that contributed to diversity under positive selection. Although such de novo gene-formation was once viewed as highly unlikely, evidence now supports the idea that protein-coding genes can indeed derive from de novo non-coding DNA. Several unequivocal examples have been published. Thus, de novo genes have been identified in Drosophila, yeast, Plasmodium, rice, mice, and primates including humans [40]. Clearly, preassembly is an evolutionary theory not only with valuable explanatory power but also with substantial empirical backing.

## 4. Conclusions

Inevitably, new questions arise with any new and speculative construct. It is unknown, for example, how environmental changes induce the appearance of new genetic constructs. Hopefully, future advances in epigenetics, a field still in its infancy, will clarify the uncertainty. It would also be advantageous to know how those particular genes and gene fragments needed for a trait are called forth from a formidable mixture of genetic potential, represented by a huge non-coding DNA library. In other words, one needs to better understand the selective passage of genetic material from the non-coded DNA repository to the active genome. Accordingly, non-coded DNA lies at the heart of what has been a mysteriously rapid acquisition of diversity in the evolution of biological complexity. Preassembly is seen to incorporate “neo-Lamarckian” notions in which new traits are acquired independent of sequential mutational modifications coupled to innumerable intermediates. Importantly, preassembly helps explain the presence of many complex features of present-day biology about which neo-Darwinism has little to say.

Andre Gide wrote: “Believe those who are seeking the truth. Doubt those who find it.” Preassembly proponents clearly belong to the former category.

## Data Availability

Not applicable.
